# Supplementary material for: Multi-modal adaptive gated mechanism for visual question answering
Source: PLoS One. 2023 Jun 28;18(6):e0287557. doi: 10.1371/journal.pone.0287557 (PMC10306234; doi:10.1371/journal.pone.0287557)
Supplement: Attachment — Submitted filename: Response to Reviewers.pdf [file pone.0287557.s001.pdf]

Manuscript ID: PONE-D-23-05435

Paper Title: Multi-modal Adaptive Gated Mechanism for Visual Question Answering

Dear Editor,

Thank you for your letter and the reviewers' comments concerning our manuscript, "Multi-modal Adaptive Gated Mechanism for Visual Question Answering" (PONE-D-23-05435). These comments are all valuable and helpful for revising and improving our manuscript and the important guiding significance to our research. We have carefully studied the comments point-by-point and revised the paper accordingly.

Thanks again for your patience and guidance!

Best regards,
Yangshuyi Xu, Lin Zhang, Xiang Shen

Reviewer's Responses to Questions

Comments to the Author

1. Is the manuscript technically sound, and do the data support the conclusions?

The manuscript must describe a technically sound piece of scientific research with data that supports the conclusions. Experiments must have been conducted rigorously, with appropriate controls, replication, and sample sizes. The conclusions must be drawn appropriately based on the data presented.

Reviewer #1: Partly

Reviewer #2: Yes

Response: Thank you for your comments and suggestions on our paper. We have carefully considered and responded to all the questions we asked you below and marked them in blue font in the manuscript for easy review.

---

2. Has the statistical analysis been performed appropriately and rigorously?

Reviewer #1: Yes

Reviewer #2: Yes

Response: We are very grateful for your affirmation of our work.

---

3. Have the authors made all data underlying the findings in their manuscript fully available?

The PLOS Data policy requires authors to make all data underlying the findings described in their manuscript fully available without restriction, with rare exception (please refer to the Data Availability Statement in the manuscript PDF file). The data should be provided as part of the manuscript or its supporting information, or deposited to a public repository. For example, in addition to summary statistics, the data points behind means, medians and

variance measures should be available. If there are restrictions on publicly sharing data—e.g. participant privacy or use of data from a third party—those must be specified.

Reviewer #1: Yes

Reviewer #2: Yes

Response: We are very grateful for your affirmation of our work.

---

4. Is the manuscript presented in an intelligible fashion and written in standard English?

PLOS ONE does not copyedit accepted manuscripts, so the language in submitted articles must be clear, correct, and unambiguous. Any typographical or grammatical errors should be corrected at revision, so please note any specific errors here.

Reviewer #1: Yes

Reviewer #2: Yes

Response: We are very grateful for your affirmation of our work.

---

5. Review Comments to the Author

Please use the space provided to explain your answers to the questions above. You may also include additional comments for the author, including concerns about dual publication, research ethics, or publication ethics. (Please upload your review as an attachment if it exceeds 20,000 characters)

Reviewer #1: Please can you explain in more detail how the gating mechanism is used to reduce noise.

Response: First, thank you for reviewing our paper and giving suggestions. We have carefully considered your questions and added new content in lines 341-353, 380-391, and 450-454 of the manuscript, marked in blue font, and we use yellow background and strikethrough to mark older content, so you can better distinguish them.

In subsection 3.2.2.1, we introduce a gating mechanism in the SAG unit. Through formula (6), the output of the multi-head self-attention mechanism X_1 and the output of the feedforward neural network X_2 can be concatenated. This step can thoroughly combine the semantic dependencies captured by the multi-head self-attention mechanism and the local features of the text captured by the feedforward network; Through formula (7) and formula (8), the information of the concatenated feature vector X_3 can be effectively limited, so that the Gated Linear Unit (GLU) in formula (9) can be used to control the information inflow of the limited feature vector adaptively; By fusing X_2 in formula (9), more low- and high-level semantic features can be retained while effectively filtering irrelevant and redundant information. Due to the short length of the question texts in the VQA task, the critical semantic features need to be fully extracted and mined. Through the above calculation process, the semantic representation ability of the feature subspace can be enhanced to better capture and represent the critical semantic information in the question texts. We marked the relevant revisions in a blue font at lines 352-365 of the manuscript.

In subsection 3.2.2.2, we introduce a gating mechanism in the SGAG unit. Through formula (12), the original feature input of the image Y and the output of the feedforward neural network Y_2 can be concatenated. This step can thoroughly combine the text information-image area interaction features captured by the multi-head self-attention mechanism and feed-forward neural network with the original image features, and then obtain rich image semantic features containing text information; Through formula (13) and formula (14), the concatenated feature vector Y_3 can be effectively limited, so that the GLU in formula (15) can be used to control the information inflow of the limited feature vector adaptively; By fusing Y_2 in formula (15), more low- and high-level semantic features can be retained while effectively filtering irrelevant and redundant information. The above calculation process can enhance the semantic representation ability of text question-guided image features. At the same time, it can improve the semantic association between text and image and better capture the critical semantic features related to text questions in image regions. We marked the relevant revisions in a blue font at lines 387-400 of the manuscript.

In Section 3.3, we introduce an adaptive gated feature fusion structure. Through formula (20), formula (21), and formula (23), deep-level and fine-grained information filtering is carried out on the preliminarily fused problem feature $\overline{X}$, image feature $\overline{Y}$, and their weighted sum feature h. Finally, through formula (24-26), the adaptive fusion of text and image feature representations can be realized so that the model can thoroughly learn the high-level modality fusion semantic feature representations. We marked the relevant revisions in a blue font at lines 447-452 of the manuscript.

We have carefully considered your constructive questions and provided detailed explanations while carefully revising the manuscript so that readers can understand our intentions.

Thank you again for reviewing our manuscript and acknowledging the relevant work we have done.

In equation 28, the left hand side of equation should be BCELoss not "N".
Response: First, we apologize for making this mistake in equation 28. After your severe suggestions and reminders, we carefully modified equation 28 accordingly. The left hand side of equation 28 should be “BCELoss” instead of “N”.

In equation 28, what does "n" represent. It should be replaced with N (as N represent number of answer categories.)
Response: First, we apologize for making this mistake in equation 28. After your severe suggestions and reminders, we double-checked Equation 28, where "n" should be "N", which we have carefully modified.

Reviewer #2: This article improves the performance of the VQA task by an introduction of a gating mechanism in intra- and inter-modal interactive learning, along with a multi-modal adaptive gated mechanism during multi-modal fusion. To better control the contribution of each modal feature to the predicted answer, the authors propose utilizing the self-attention gated unit and the self-guided attention gated unit. Experiment results on the VQA2.0 and GQA benchmark datasets show the relevence of the proposed model. In general, the contribution of this work is significant. I recommend this work to be published.

Response: We are very grateful that you took the pains to review our manuscript and were able to acknowledge our relevant work in the manuscript.
